# Interference with KCTD9 inhibits NK cell activation and ameliorates fulminant liver failure in mice

**DOI:** 10.1186/s12865-018-0256-x

**Published:** 2018-06-25

**Authors:** Xiaoping Zhang, Lin Zhu, Yaoyong Zhou, Aichao Shi, Hongwu Wang, Meifang Han, Xiaoyang Wan, Semvua Bukheti Kilonzo, Xiaoping Luo, Tao Chen, Qin Ning

**Affiliations:** 1 Institute of Infectious Disease, Tongji Hospital of Tongji Medical College, Huazhong University of Science and Technology, # 1095 Jiefang Avenue, Wuhan, 430030 People’s Republic of China; 2 Department of Infectious Disease, Tongji Hospital of Tongji Medical College, Huazhong University of Science and Technology, Wuhan, China; 3 Department of Pediatric Disease, Tongji Hospital of Tongji Medical College, Huazhong University of Science and Technology, Wuhan, China

**Keywords:** KCTD9, Short hairpin RNA, NK cell, Liver failure, MHV-3

## Abstract

**Background:**

Potassium channel tetramerisation domain containing 9 (KCTD9), a member of KCTD family with a DNA-like pentapeptide repeat domain, was found to be increased particularly in NK cells of patients with HBV-induced acute-on-chronic liver failure (HBV-ACLF) and experimental viral fulminant hepatitis. Knockdown of KCTD9 in immortalized NK cells inhibits cytokines production and cytotoxicity. As NK cell activation was shown to exacerbate liver damage in viral fulminant hepatitis, we propose that target inhibition of KCTD9 may prohibit NK cells activity and thus ameliorate liver damage in viral fulminant hepatitis.

**Result:**

Hydrodynamic delivery of plasmid expressing short-hairpin RNA against KCTD9 resulted in impaired NK cells function as demonstrated by reduced cytokine production and cytotoxicity, and ameliorated liver injury as manifested by improved liver histology and survival rate. In contrast, delivery of plasmid expressing KCTD9 led to deteriorated disease progression.

**Conclusion:**

Interference with KCTD9 expression exert beneficial effect in viral fulminant hepatitis therapy. Such effect may be mediated by impairment of NK cell activation.

**Electronic supplementary material:**

The online version of this article (10.1186/s12865-018-0256-x) contains supplementary material, which is available to authorized users.

## Background

More than 350 million people worldwide suffer from chronic Hepatitis B virus (HBV) infection. Chronic HBV infection results in a wide spectrum of clinical presentations, among which acute-on-chronic liver failure (ACLF) is one of the most severe manifestation [1]. The mortality of HBV-induced acute-on-chronic liver failure (HBV-ACLF) is 50–70% in cases lacking immediate liver transplantation [2]. Limitation of treatment strategies leads to ineffectiveness of physical therapies.

NK cells, an innate immune cell subset with immune regulatory property as well as cytotoxicity, has been revealed to be involved in liver damage in multiple liver disease such as autoimmune disease, hepatitis, and fibrosis [3–5]. Our previous work demonstrated that increased killing of hepatic NK cells, and infiltration of peripheral NK cells into the liver, contributed to hepatocyte injury through Fas/FasL and NKG2D/NKG2DL pathways in patients with HBV-ACLF and in mice of murine hepatitis virus strain 3 (MHV-3)-induced fulminant hepatitis failure (FHF). Depletion of NK cells facilitated the liver function recovery from the severe phase of acute or chronic infection [6].

Potassium channel tetramerisation domain containing 9 (KCTD9), a member of KCTD family, contains a unique eukaryotes-specific DUF3354 domain and pentapeptide repeat which confers bacterial resistance to quinolones by mimicking structure and surface charge of DNA [7]. Pentapeptide repeats also interacts with DNA gyrase and inhibits gyrase activity in bacterial [8, 9]. However, the function of DUF3354 domain is not clear. Until recently, evidence regarding KCTD9 functions is almost lacking. One evidence linking to KCTD9 that its interaction with MED20 serves as scaffold and assembly for conferring bridging gene specific regulatory apparatus to basal RNA polymerase II transcription machinery [9]. Another evidence for the function of KCTD9 is that its orthologue (FIP2) in plants interacts with FORMIN1 for orchestration of nuclear export machinery [10]. Structure analysis reveals that KCTD9 acts as adaptor bridging substrate proteins and Cul3, an E3 ligase that ubiquinates substrate proteins for proteasomal degradation [11].

Recently, we newly identified more abundant expression of KCTD9 in peripheral and hepatic NK cells and T cells from patients with HBV-ACLF relative to mild chronic hepatitis B (CHB) patients [12]. Moreover, enhanced KCTD9 expression in peripheral and hepatic NK cells was positively correlated with the severity of liver injury in HBV-ACLF patients [13]. In vitro, the KCTD9 overexpression in NK92 cells resulted in increase of CD69 expression, elevated cytotoxicity to HepG 2.2.15 cell line, and marked decrease of the NKG2A receptor suggestive of enhanced activity NK cells [12]. These results also imply potential engagement of KCTD9 expression in liver damage by activating NK cells.

To evaluate our hypothesis that KCTD9 may be adopted as an interfering target to acute viral liver failure, short hairpin RNAs (shRNAs) targeting mouse KCTD9 specifically was constructed and administrated via hydrodynamic delivery to mice infected with MHV-3. Our data showed that effective inhibition of liver KCTD9 expression by mKCTD9 shRNAs significantly reduced cytokines production and cytotoxicity of hepatic NK cells, and slowed down FHF progression as manifested by increased survival rate and alleviated liver injury. Our study provides insights for targeted treatment of virus-induced liver failure by modulating hepatic NK cells function.

## Methods

### MHV-3 induced fulminant hepatitis Balb/cJ mouse model

MHV-3 was obtained from the American Type Culture Collection (Manassas, VA), plaque purified on monolayer of delayed brain tumor cells (DBT), and titer tested on L2 cells according to a standard plaque assay [14, 15]. DBT cells and L2 were provided generously by Dr. Gary A Levy (Toronto General Hospital, Toronto). Female BALB/cJ mice at 6–8 weeks old, weighed 18-20 g, were purchased from HFK Bioscience (Beijing). Viral fulminant hepatic failure was established by intraperitoneal injection of either 20 or 100PFU of MHV-3. In detail, MHV-3 was diluted in sterile PBS at a concentration of 100PFU/ml or 500 PFU/ml. Mice were injected intraperitoneally with MHV-3 (20 PFU or 100 PFU per mouse) in a total volume of 200 μl. Mice were anaesthetized by exposure to 0.08 ml/l aether (1.9%) and sacrificed by cervical dislocation for sample collection at 48 h post viral injection. Animal care and procedures on animal were in compliance with the guidelines outlined in the Guide for the Care and Use of Laboratory Animals and approved by the Committees on Animal Experimentation, Tongji Hospital, Tongji Medical College, Huazhong University of Science and Technology.

### Plasmids and hydrodynamic delivery

Independent shRNAs against mouse KCTD9 locus (+ 411 bp and + 801 bp) were designed according to the literature previously [16]. The fragment was cloned into shRNA expressing plasmid with U6 promoter at the upstream as we previous described [17]. The fragment spanning U6 and shRNA was amplified and then subcloned into the PMSCV-neo expression vector to generate pMSCV-mKCTD9-shRNA. A scramble shRNA was also designed and cloned into expressing vector in parallel as control. With regard to KCTD9 overexpressing construct, the full-length open reading frame of the mouse KCTD9 cDNA was amplified with Not I and Hind III recognition site hanging at 5′ and 3′ end respectively. PCR product was purified, enzyme-digested, and cloned into pcDNA3.1 empty construct (Invitrogen, CA, USA) to generate KCTD9-expressing plasmid pcDNA3.1-mKCTD9. The constructs were verified by sequencing. Primers sequence were shown in Table 1. Hydrodynamic delivery of plasmids was conducted at 24 h before and after MHV3 infection, respectively by tail-vein injection of 200 μg/200 μl per mice. Eighteen mice was included in each cohort. The injection time was controlled in 15- 20s per mice. The injections were completed within 2 h.

**Table 1:** Primers used in mKCTD9 cloning, shRNA and quantitative PCR

Gene	Primer
Mouse KCTD9 (for cloning)	anti-sense 5’ ATAAGAATGCGGCCGCGCACCTCTGGGAACAAGC 3’
	sense 5’ CCCAAGCTTGTGCGTGCGTGAGGAGTGT 3’
mKCTD9-shRNA1	anti-sense 5’ AAGCTTAAAAATTGACCGAAGTCCTGAGTATCTCTTGAATACTCAGGACTTCGGTCAAAAACAAGGCTTTTC 3’
mKCTD9-shRNA2	anti-sense 5‘AAGCTTAAAAAGCCAGATGACAGGGATTAATCTCTTGAATTAATCCCTGTCATCTGGCAAACAAGGCTTTTC 3’
shRNA-Negative Control	anti-sense 5’ AAGCTTAAAAAGACGCAACGGAAGTGTTAATCTCTTGAATTAACACTTCCGTTGCGTCAAACAAGGCTTTTC 3’
mKCTD9-shRNA	sense 5’ GTAGGATCCATCCGACGCCGCCATCTC 3’
mKCTD9 (for qPCR)	anti-sense 5’ ACGATACTTCACAACAACAAGG 3’
	sense 5’ CCTGGCTTCTTCTAGCACAC 3’
GAPDH (for qPCR)	anti-sense 5’ CGGATTTGGTCGTATTGGG 3’
	sense 5’ CTCGCTCCTGGAAGATGG 3’

### Cell lines and transfection

Chinese hamster ovary (CHO) cells, purchased form Cell Resource Center for Biomedical Research, were cultured in six-well plates until 50–80% confluence. Then plasmid generated above were transfected using Lipofectamine 2000 (Invitrogen, Carlsbad, CA) according to the manufacturer’s protocol. Cells were harvested at 48 h after transfection for either protein collection or RNA isolation. Western blot and qPCR were conducted to detect shRNA efficiency and KCTD9 overexpressing.

### Preparation of mouse liver mononuclear cells

Liver tissue and PBMCs were collected at 48 h post MHV-3 infection. Mononuclear cells were isolated from livers by passing tissue through a 200-gauge stainless steel mesh in RPMI 1640 medium (Gibco BRL, Grand Island, NY). The cell suspension was then centrifuged at 500 g for 5 min, and the supernatant was discarded. The cell pellet was resuspended in 40% percoll (Sigma-Aldrich, St. Louis, MO) in RPMI 1640 medium via vigorous vortexing. The cell suspension was gently overlaid onto 70% percoll and centrifuged for 20 min at 750 g at room temperature. Mononuclear cells were collected from the interphase and then washed twice in PBS containing 5% fetal bovine serum. The degree of contamination by Kupffer cells and hepatocytes was minimal.

### Quantitative PCR

cDNA was synthesized from 1 μg RNA samples and diluted for 10 times. Quantitative PCR (qPCR) was performed using SYBR Green Mastermix (Toyobo, Osaka, Japan) to detect KCTD9 expression. The relative value was calculated with standard methodology of 2^-△△Ct^. GAPDH was set as internal control. The primers was listed in Table 1.

### Western blot analysis

Twenty micrograms of protein from cell extract was boiled for 5 min in 2× sodium dodecyl sulfate (SDS) buffer containing 20% dithiothreitol (DTT) and resolved by SDS-polyacrylamide gel electrophoresis (PAGE). The resolved proteins were transferred to a PVDF membrane. The membrane was blocked in 5% nonfat milk and 0.05% Tween 20 in PBS for 2 h at room temperature with shaking and then probed for 1 h at room temperature with shaking using an anti-KCTD9 polyclonal antibody in dilution of 1:2000 [12]. The membranes were rinsed twice with PBS and washed 6 times with 0.05% Tween 20 in PBS for 5 min with shaking. The membrane was probed with a horseradish peroxidase-labeled goat anti-rabbit secondary antibody (Santa Cruz Biotechnology, Santa Cruz, CA) for 1 h with shaking before being rinsed twice with PBS and washed 6 times in 0.05% Tween 20 in PBS for 5 min. The blots were developed by chemiluminescence for 2 min and exposed to X-Omat blue film (PerkinElmer Life and Analytical Sciences, Boston, MA) for 1 s to 30 min.

### Immunocytochemistry and immunohistochemistry

Freshly isolated PBMCs were overlaid on polylysine treated slides, fixed with 4 °C acetone, and then air-dried. After blocking for nonspecific staining, the following process was conducted using an immunohistochemistry kit (SP9001/SP9002; Zhongshan Biotechnology, Beijing, China) according to the manufacturer’s instructions. In brief, deparaffinized sections were immunologically stained with optimal dilutions of rabbit anti-human KCTD9 polyclonal antibody at 37 °C for 60 min. The sections were then incubated with HRP-conjugated goat anti-rabbit mAb (BD Biosciences, San Diego, CA) secondary antibody at 37 °C for 30 min. As negative controls, nonimmunized rabbit IgG was used as the primary antibody. Sections were analyzed under a light microscope (Carl Zeiss, Thornwood, NY). The same protocol was used to detect KCTD9 expression in human and mouse liver sections.

### Flow cytometry

Fluorescence-conjugated antibodies were purchased from eBioscience. Antibodies are: PerCP-Cy5.5-conjugated CD3 (clone 500A2), APC-Cy7-conjugated CD4 (clone L3 T4), APC-conjugated CD8 (Ly-3), PE-conjugated CD69 (clone H1.2F3). Antibody stained was conducted according to the manufacture’s instruction. Briefly, mononuclear cells were treated with Fc blocker (anti-CD16/CD32, eBioscience) for 10 min at 4 °C and washed with PBS twice. Then, cells were stained with surface antibody cocktail (CD3- PerCP-Cy5.5, CD4- APC-Cy7, CD8-APC, CD69-PE) at 4 °C for 30 min, washed twice with PBS. For intracellular KCTD9 staining, mononuclear cells stained with surfaced antibodies as described above, and followed by fixation and permeabilization (BD bioscience, 554,715) for 20 min at 4 °C. Cells were washed with PBS twice and stained with KCTD9 antibody at room temperature for 1 h. FITC-conjugated goat anti-rabbit polyclonal IgG secondary antibody was used for visualization. Normal rabbit polyclonal IgG (eBioscience, San Diego, CA) staining was used as a negative control. Cells were acquired using a Canto-II flowcytometer (Becton, Dickinson and Company, Franklin Lakes, NJ).

### Cytotoxic assays

The hepatic NK cells of infected BALB/cJ mice were isolated using a MACS separation kit (Miltenyi Biotech, Germany) and cultured in RPMI 1640 medium with 10% fetal bovine serum (FBS). The remaining hepatocytes were set as the target. The target-cell only group cultured with 100 μl lysis solution (Fermentas, Glen Burnie, MD) for an hour as the maxium-releasing group and the natural cultured cell group was set as the spontaneous-releasing group. ALT was quantitated using an Automatic Chemistry Analyzer (Hitachi, Tokyo, Japan). The cytotoxicity was defined as ratio of (experimental releasing - spontaneous releasing) / (maxium releasing - spontaneous releasing) * 100%. Triplicate assays were conducted.

### Statistical analysis

Statistical significance was determined using the Student’s t test (2-tailed) or ANOVA, as appropriate. All statistical analyses were performed with SPSS v.11 (SPSS, Chicago, IL). A *p* value of less than 0.05 was considered statistically significant. All results are presented as mean ± SEM.

## Results

### KCTD9 expression significantly elevated in intrahepatic lymphocytes of MHV-3-FHF mice

**Fig. 1:**
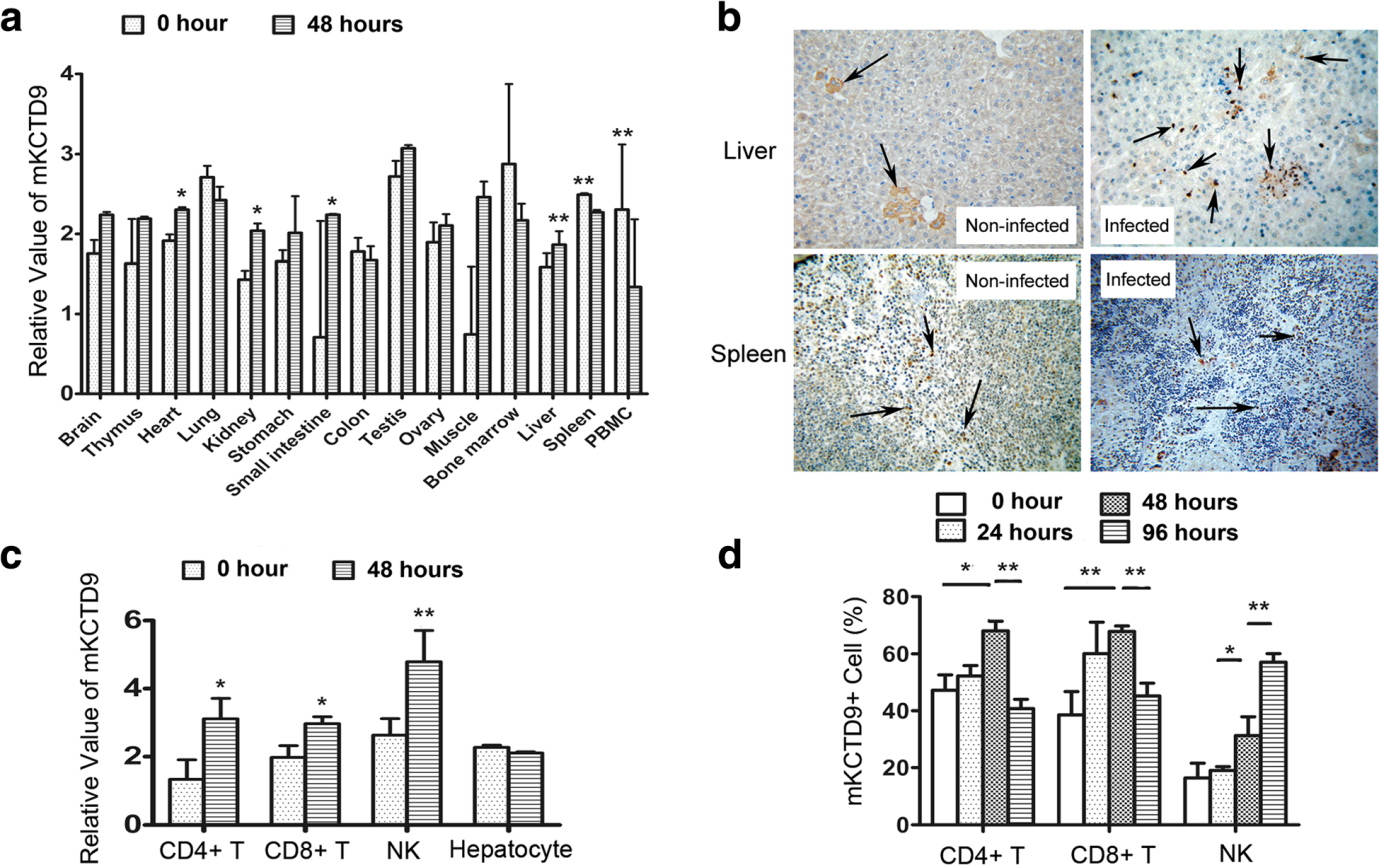
Elevated KCTD9 expression bothin liver tissue and hepatic NK cells in MHV-3-FHF mouse model. **a** KCTD9 expression in liver, heart, kidney, spleen, PBMC was determined in Balb/cJ mice with or without infection of 100 PUF of MHV3. **b** The expression of KCTD9 protein in liver and spleen 48 h after MHV-3 infection. Magnification: 400 X. **c** mKCTD9 mRNA levels in hepatic NK cell, CD4^+^ T cell, CD8^+^ T cell and hepatocyte isolated from Balb/cJ mice with or without MHV-3 infection. **d** The FACS assay showed that Percentage of hepatic CD4^+^ T cells and CD8^+^ T cells expressing KCTD9 in mice with or without MHV-3 infection for 24, 48, 72 and 96 h. *p < 0.05, **p < 0.01, Means ± SEM of 3 independent experiments were represented

To evaluate the pathological resemblance of MHV-3-FHF mice model to human HBV-ACLF disease, the expressions of KCTD9 in a variety of organs and tissues from MHV-3-FHF mice model, including the liver, heart, kidney, spleen, and PBMCs were measured at 48 h after MHV-3 infection when over 80% of mice were alive (Additional file 1: Figure S1). KCTD9 was remarkably up-regulated in the liver (*p* < 0.01), heart (*p* < 0.05), and kidney (p < 0.05) but significantly down-regulated in the spleen (p < 0.01) and PBMCs (p < 0.01) (Fig. 1a, Table 2). Dominant expression of KCTD9 was restricted in the infiltrating cells and was enhanced after infection in the liver, while basal expression of KCTD9 was observed but almost unaltered in the hepatocytes (Fig. 1b). In the spleen, the expression of KCTD9 was moderate in most of lymphocytes at physiological settings, and was up-regulated in individual cells after MHV-3 infection although the number of lymphocytes expressing KCTD9 decreased (Fig. 1b), suggesting mobilization of lymphocytes in to peripheral tissues (Fig. 1b). This hints was documented by KCTD9 expression was decreased in the spleen and PBMCs, but increased in the liver at mRNA levels from gross tissues (Fig.1a, Table 2). Beside, KCTD9 expression was also up-regulated in the kidney, hear, and small intestine based on PCR result thought such data was rough (Fig.1a), suggesting inflammation occurred in such tissues, a phenomenon resembling progression of viral acute liver failure in patients. Moreover, the levels of KCTD9 mRNA was increased in hepatic NK cells, CD4^+^ T cells and CD8^+^ T cells by 48 h of infection, without significant difference in hepatocytes (Fig. 1c). The percentage of hepatic NK cells expressing KCTD9 protein was persistently elevated until the death of the mice (Fig. 1d). These data suggested KCTD9 was predominant expressed in lymphocytes and particularly induced following viral infection.

**Table 2:** Relative vaule of mKCTD9 mRNA level from real time PCR results corresponding to Fig. 1a

	Brain	Thymus	Heart	Lung	Kidney	Stomach	Small intestine	
0 h	2.455 ± 0.170	2.331 ± 0.558	2.615 ± 0.079	3.411 ± 0.142	2.131 ± 0.111	2.358 ± 0.140	2.409 ± 0.395	
48 h	2.938 ± 0.306	2.890 ± 0.027	2.804 ± 0.030	3.123 ± 0.168	2.541 ± 0.091	2.713 ± 0.460	2.940 ± 0.012	
t value	−2.392	−1.734	−3.893	2.251	−3.933	−1.283	−2.325	
p value	0.075	0.225	0.018	0.088	0.017	0.269	0.081	
	Colon	Testis	Ovary	Muscle	Bone Marrow	Liver	Spleen	PBMC
0 h	2.480 ± 0.172	3.420 ± 0.195	2.596 ± 0.249	1.945 ± 0.142	3.575 ± 0.999	2.118 ± 0.054	2.193 ± 0.017	1.331 ± 0.575
48 h	2.373 ± 0.175	3.774 ± 0.140	2.805 ± 0.144	2.461 ± 0.197	2.870 ± 0.209	2.786 ± 0.389	1.971 ± 0.030	3.112 ± 0.602
t value	1.002	−2.563	−1.257	−3.633	1.199	−2.946	11.195	−3.706
p value	0.365	0.062	0.277	0.022	0.297	0.042	< 0.001	0.021

### shRNAs induced KCTD9 silence in vitro

**Fig. 2:**
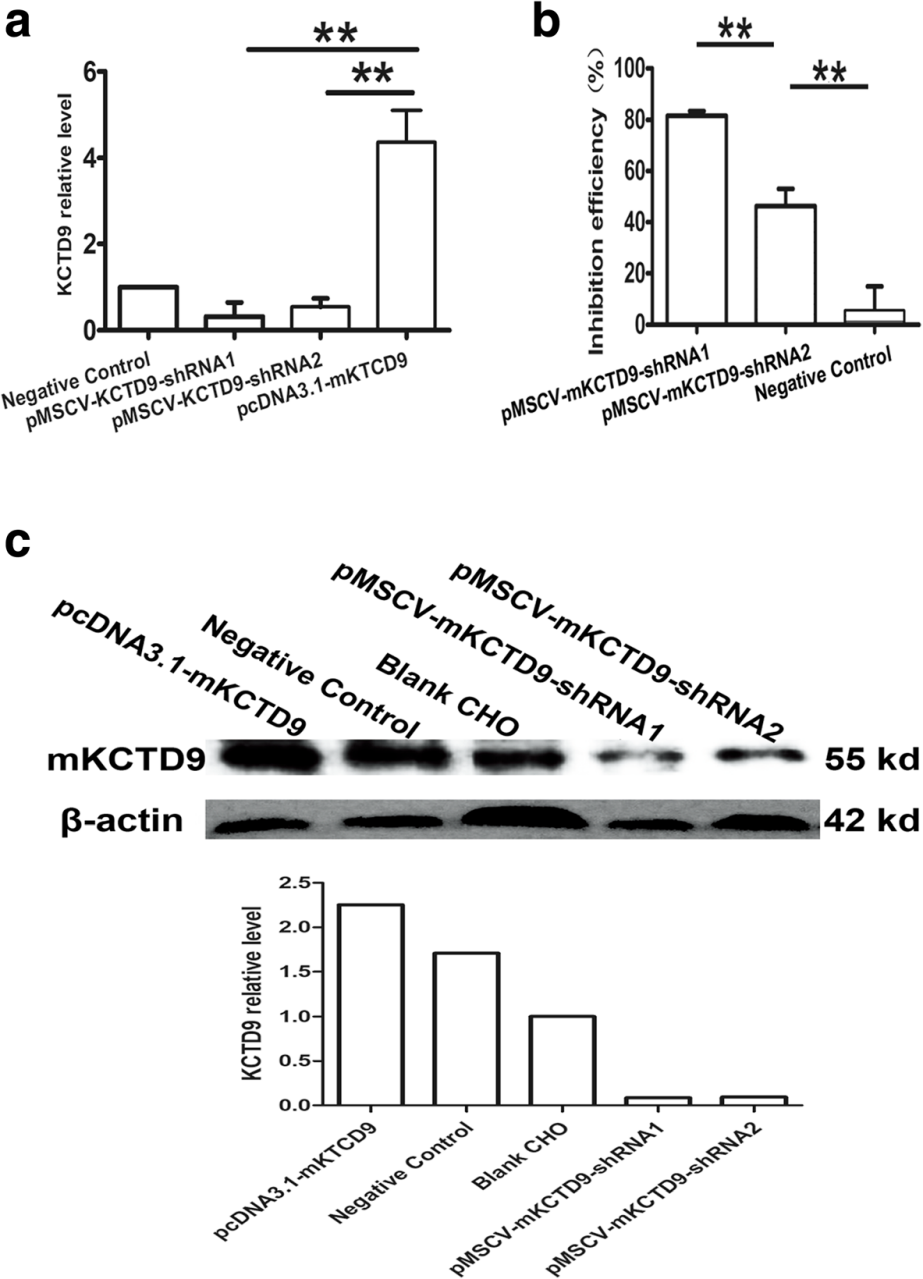
mKCTD9 shRNAs inhibited mKCTD9 expression in vitro. **a** Relative fold of mKCTD9 mRNA in CHO cell transfected with either shRNAs against mKCTD9, pcDNA3.1-mKCTD9, as well as scramble control. **b** inhibition efficiency or shRNAs against mKCTD9. **c** West bolt analysis of mKCTD9 expression in CHO cells transfected with pcDNA-mKCTD9, pMSCV-mKCTD9-shRNA1, pMSCV-mKCTD9-shRNA2, pMSCV-scramble shRNA (Negative Control as indicated in the figure); relative value of mKCTD9 protein levels were (from left to right): 2.25, 1.71, 1.0, 0.86, 0.96. **p < 0.01

In order to measure the efficacy of ectopic expression and gene silencing of KCTD9, plasmids such as pcDNA3.1-mKCTD9, pMSCV-mKCTD9-shRNAs as well as negative control were transfected into CHO cell line. The expression of KCTD9 expression was significantly increased in cells transfected with pcDNA3.1-mKCTD9, and decreased in cells transfected with pMSCV-mKCTD9-shRNAs in both mRNA and proteins levels (Fig.2a-c). The mRNA level of KCTD9 was suppressed by almost 90% by shRNA1 (81.8 ± 2.0%) and 50% (46.2 ± 6.6%) by shRNA2, respectively (Fig.2a). The protein level of KCTD9 was also declined to a great extent by either shRNA1 or shRNA2 (Fig. 2c). Protein level of KCDT9 was increased to almost 1.4 by transfection of pcDNA3.1-mKCTD9 (Fig.2c), which might result from high level of basal expression of KCTD9 expression in CHO cells. These data suggests effectiveness of up-regulation and silence of KCTD9 expression can be achieved by transient transfusion of mKCTD9- and shRNA-expressing plasmids, respectively.

### Interference of KCTD9 resulted in increased survival rate of mice of MHV-3 infection

**Fig. 3:**
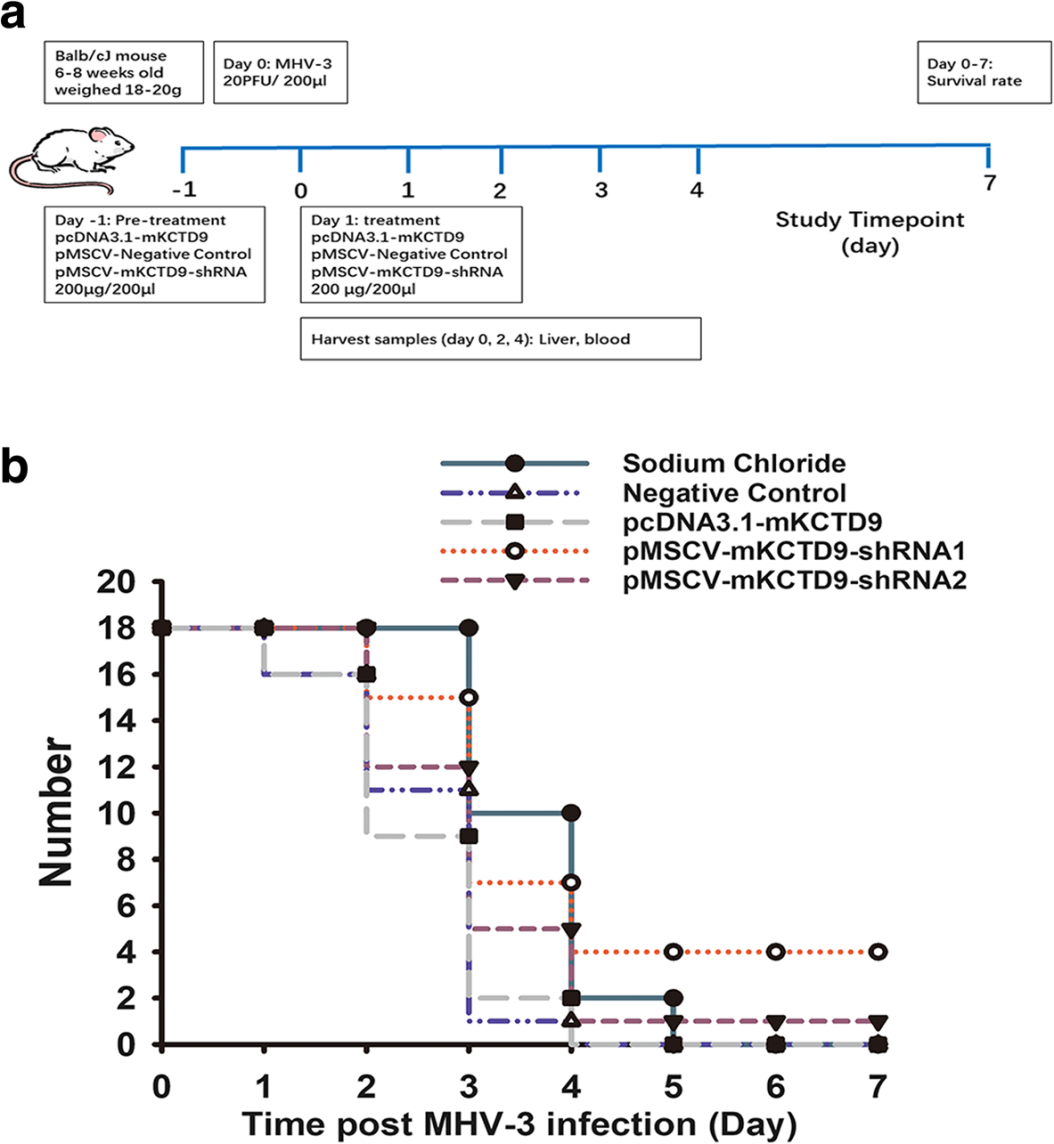
Increased survival in mice of mKCTD9 interference. **a** Schematic view of hydrodynamic delivery of plasmid and tissue collection. Individual plasmid were injected into mice with 200 μg plasmid in 200 μl saline by tail-vein injection at 24 h before and after MHV-3 infection. 20 PFU of MHV3 were injected intraperitoneally in each mice. Tissues were collected at indicated time point specifically at 24, 48 and 72 h post MHV3 infection. **b** Time of infected mice which were injected with pMSCV-mKCTD9-shRNA1, pMSCV-mKCTD9-shRNA2, pcDNA3.1-mKCTD9, Negative Control plasmid and saline, was calculated until death. Eighteen mice were pooled together in each cohort

To further investigate the biological effects in vivo, pMSCV-mKCTD9-shRNAs, mKCTD9-expressing plasmid, as well as the Negative Control plasmid was administered twice by hydrodynamic delivery into mice (Fig.3a). As almost 80% of mice died within 48 to 72 h post infection of 100 PFU of MHV3 (Additional file 1: Figure S1), we infected mice with 20 PFU of MHV-3, which manifest less rapid progression to death (Additional file 1: Figure S1), to look at pathological effect of these plasmid on disease progression. Mice delivered with negative control plasmid showed rapid loss (7/18 mice died within 48 h, 10/18 mice died between 72 and 96 h; 1/18 died at 96 h; time of MHV-3 infection as 0), likely to be worse than that injected with saline (8/18 died between 72 and 96 h, 8/10 died between 96 and 108 h, 2 died by 120 h) (Fig. 3b). However, Mice delivered with pMSCV-mKCTD9-shRNA1 exhibited less rapid progression than negative control cohort as evident by 2/18 mice loss within 48, 8/18 mice loss between 48 and 72 h, 4/18 loss between 72 and 96 h with 4 survived (Fig. 3b). Mice delivered with pMSCV-mKCTD9-shRNA2 showed similar result by mice loss of 6/18 mice within 48 h, 7/18 between 48 and 96 h, 4/18 between 96 and 108 h, and 1 18 survived (Fig. 3b). Interestingly, mice delivered with pcDNA3.1-mKCTD9 more rapid progression of disease than that of negative control by mice loss of 9/18 within 48 h, 7/18 between 48 and 72 h, and 1/18 by 96 h after infection (Fig. 3b). These data suggest mKCTD9 interference ameliorates liver damage and promotes mice survival in FHF. Vice versa, overexpression of mKCTD9 exacerbates liver damage and prompt disease progress of FHF.

### Interference of KCTD9 resulted in improved liver function and histologic recovery in mice with MHV-3-FHF

**Fig. 4:**
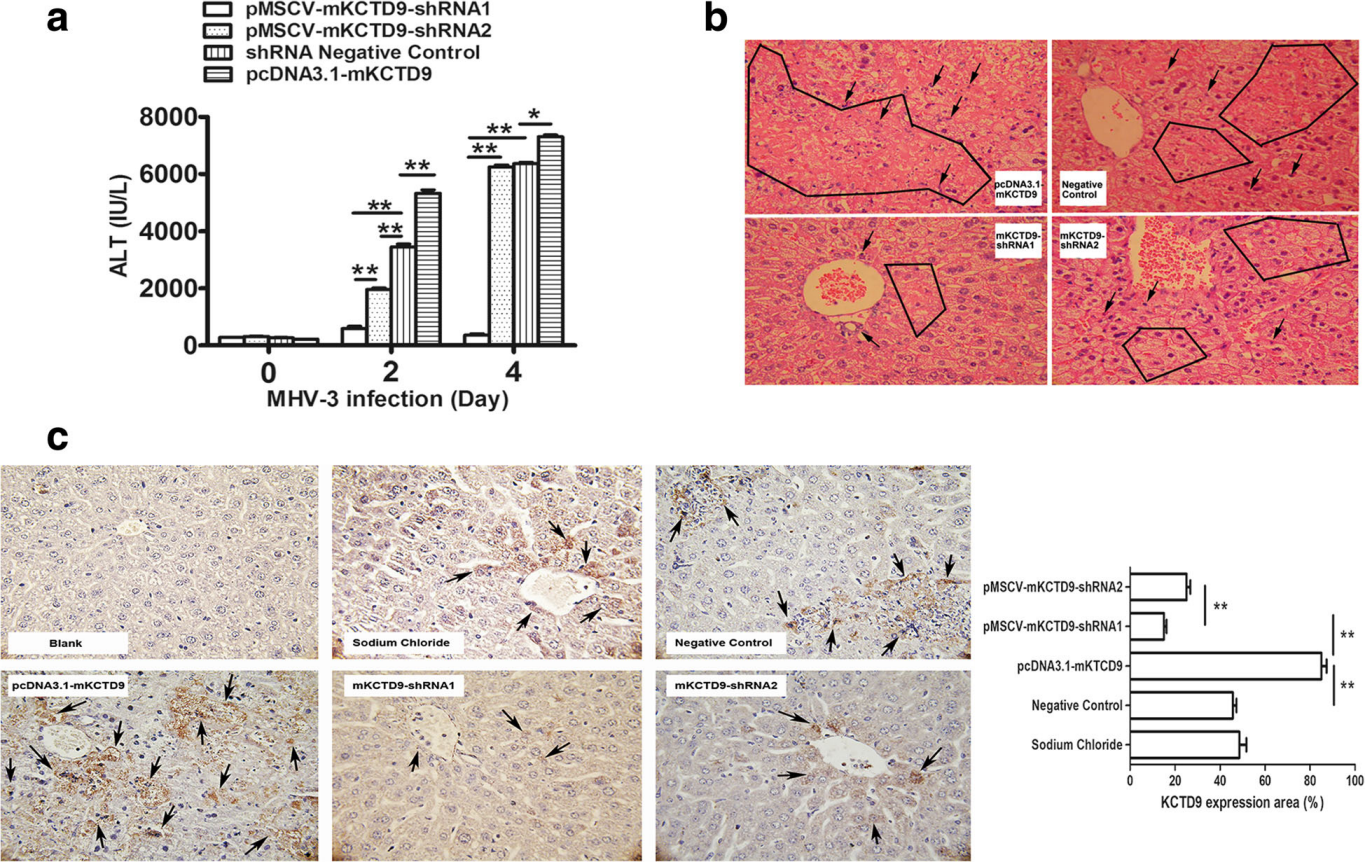
KCTD9 shRNA inhibited KCTD9 expression and ameliorates MHV-3 induced fulminant hepatitis in vivo. **a** ALT levels of infected mice delivered with distinct plasmid at 24 h,48 h and 108 h after MHV-3 infection. **b** Hematoxylin and eosin (H&E) staining of livers sections from mice 4 days post MHV-3 infection. Black arrow indicated the inflammatory cells area within black lines indicated liver necrosis. Magnification: 400 X. **c** Immunochemistry staining against mKCTD9 on liver sections of mice injected with various plasmid at 36 h after MHV-3 infection. Blank points to IgG stained section; The brown staining area indicates signals of KCTD9 protein. Magnification: 400 X. *p < 0.05, **p < 0.01, Means ± SEM of 5 to 7 independent experiments were represented

To better understand the mechanism underlying the biological effects of the pMSCV-mKCTD9 shRNAs, a time course study of serum ALT levels and liver pathohistology was performed after MHV-3 infection. Serum ALT levels in the pMSCV-mKCTD9-shRNA cohorts were significantly decreased compared to that in the Negative Control group at 48 and 96 h of MHV-3 infection (Fig. 4a), with shRNA1 cohorts declined to greater extent when compared with shRNA2 and negative control cohorts. Expectedly, mice delivered with pcDNA3.1-mKCTD9 exhibited higher levels of ALT compared to controls. In consistent with ALT levels, liver damage from each cohort of mice showed various extend of necrosis in the liver, with large area, moderate area, and small area of necrosis in mice delivered with pcDNA3.1-mKCTD9, negative control plasmid, and pMSCV-KCTD9-shRNA, respectively (Fig. 4b). Massive lymphocytes infiltration in pcDNA3.1-mKCTD9 cohorts, and less infiltrates in mice delivered with KCTD9-shRNAs expressing plasmids, was also observed (Fig. 4b). To confirm the results by effectiveness of plasmid, detection of KCTD9 expression was performed on liver sections. Enhanced mKCTD9 expression could be observed in lymphocytes specifically from sections from mice treated with pcDNA3.1-mKCTD9 (Fig. 4c). In contrast, the expression of mKCTD9 was relieved on sections from mice treated with shRNAs when compared with negative controls (Fig. 4c). These data suggests hydrodynamic delivery of mKCTD9-shRNA significantly relieved liver injury and promotes survival of mice of FHF, while ectopic expression of mKCTD9 exacerbates liver damage. Importantly, such effect was achieved by modulation of mKCTD9 expression by delivery of plasmids into lymphocytes.

### KCTD9 shRNA interference reduced target gene expression and hepatic NK cells activation and cytotoxicity

**Fig. 5:**
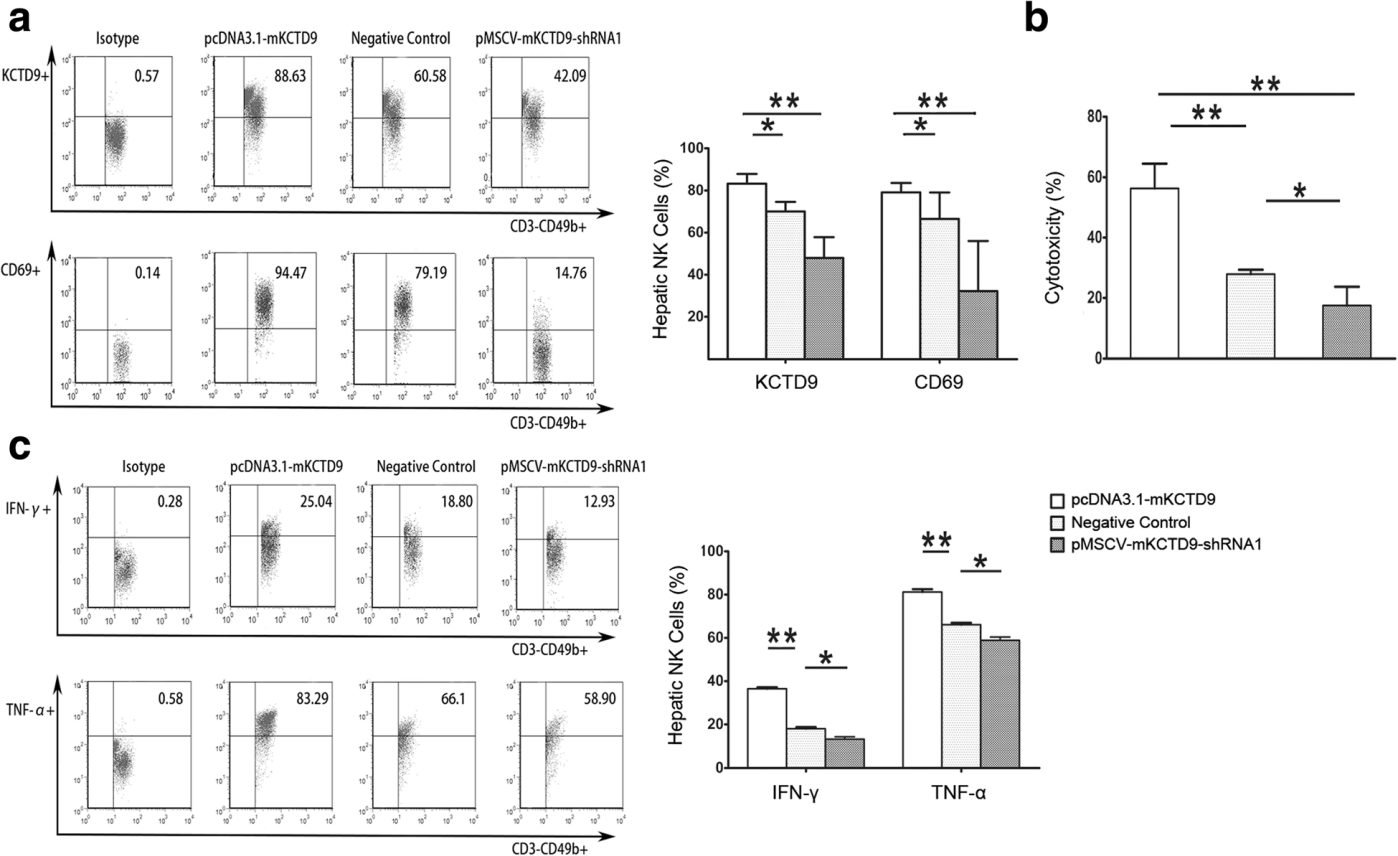
mKCTD9 interference inhibited hepatic NK cells activation and function. **a** Frequency of NK cells expressing KCTD9 as well as CD69 positive NK cells in liver were measured by FACS. **b** The Cytotoxicity of isolated hepatic NK cells from MHV-3 infected mice which were treated with mKCTD9 shRNA expressing plasmids or negative control. Cytotoxicity of NK cells calculated as following: (Experimental ALT – Spontaneous release ALT) / (Maximum ALT – Spontaneous release ALT) * 100%. **c** Frequency of IFN-γ and TNF-α expressing hepatic NK cells were measured by Flow cytometry. *p < 0.05, **p < 0.01, Means ± SEM of 3 to 5 independent experiments were represented

mKCTD9 was predominantly expressed in NK cells, and to lesser extent in T cells as we previous reported [12]. Multiple evidence of FHF by MHV3 infection suggested innate immunity impose a most important role in the pathogenesis as rapid progression of the disease. Considering the above, we dissected effect of KCTD9 particularly in NK cells in those cohorts of mice. Mice delivered with pcDNA3.1-mKCTD9 exhibited a large portion (around 80%) of hepatic NK cells expressing mKCTD9, however, a small portion of NK cells (around 40%) expressing mKCTD9 in KCTD9-shRNA1 treated mice when compared with negative cohorts (40%) (Fig. 5a-b). CD69 expression, a hallmark of NK cell activation, was also observed to be expressed in most NK cells in mice treated with pcDNA3.1-mKCTD9, and smaller population of NK cells in KCTD9-shRNA1 cohort when compared to negative controls (Fig. 5a-b). Functionally, NK cells form pcDNA3.1-mKCTD9 cohorts exhibited more enhanced cytokines production such as IFN-γ and TNF-α (Fig. 5c), and enhanced cytotoxicity to hepatocytes in vitro (Fig. 5b), as compared negative controls. In contrast, NK cells from mice delivered with pMSCV-mKCTD9-shRNA1 exhibited less cytokines production and cytotoxicity as compared to negative controls (Fig. 5b-c). These data suggests that ectopic mKCTD9 expression enhanced NK cells activity which suppression of mKCTD9 attenuated NK cells activation in vitro.

## Discussion

In last decade, a number of evidences investigate pathogenesis of liver injury in human diseases and animal models using strategy of hydrodynamic delivery of plasmids [18–21] . Specifically, RNA interference (RNAi) has also been employed as an effective way to introduce gene silencing in acute viral hepatitis and ischemia-reperfusion injury [22, 23]. Nowadays, adenoviral, retroviral, lentiviral and adeno-associated viral (AAV) vectors were developed to effectively introduce gene fragments into different types of cell [24]. However, host immune response against pseudo-virus limited their wide applications in vivo [25]. Higher dose of shRNA expressed by exogenous vectors may elicits cytotoxicity [26]. In our experiments, hydrodynamic delivery of negative control plasmid introduced liver injury as demonstrated by increased progression of FHF compared to those injected with saline (Fig. 3b). Acute liver injury is introduced after hydrodynamic injection of plasmid with peak ALT level (200 U/ml) at 48 h [27], though not high when compared with ALT levels of FHF. Such effect may explain why mice injected with negative control plasmid exhibited a trend of more rapid progression to death than mice injected with saline.

Transfusion of plasmid into host cells is not limited in specific cell types. The expression pattern of KCTD9 that robust expression is maintained in lymphocytes with enhanced expression after viral infection. However, such phenomenon is not observed in hepatocytes though minimal expression of KCTD is maintained. From our previous research as well as current work, mKCTD9 is predominantly expressed and robustly induced in lymphocytes, particular NK cells in the liver. The differential induction of KCTD9 expression between T cells and NK cells has been depicted HBV-ACLF, with more abundant expression in NK cells than that in CD4 T and CD8 T cells [12]. It is noteworthy that MHV3 infection prompt rapid progression of liver damage that fails into hepatitic failure. It accepted that innate immune response, but not adaptive immunity dominate immune-mediated liver damage in acute viral hepatitis because of rapid response by innate immunity [28]. Focus on NK cells which imposes substantial function on liver damage and deleterious effect on liver damage, may be potent target host cells of plasmid for FHF progression.

KCTD9 proteins are localized at nucleus as well as cytoplasm in hepatic NK cells, which was similar with that in NK cells from patients with HBV-ACLF [12]. These data suggest mKCTD9 may be involved in transcriptional activation or signaling transduction for activation of immune cells, particularly NK cells. The observation that direct interact of KCTD9 with Cul3, an E3 ligase [7], imply a potential role in mediating proteasomal degradation for turnover of NK cell function. Bioinformatics (STRING 8.2) prediction suggests that KCTD9 interacts with SH2 domain-containing adapter protein B (SHB) which is known as adaptor protein in T-cell antigen receptor (TCR) signaling [29], interleukin-2 signaling [30], and apoptosis [31]. SH2 domain (SRC homology 2 domain) contains tyrosine kinase activity and is involved in many component of signal cascade including SHB for immune cell activation in but not restricted to T cells and NK cells upon stimulation [32, 33]. These cues also suggest potential engagement of KCTD9 in T cell activation as TCR signaling is required for T cell maturation and activation in term of antigen recognition and cytokines production [34]. IL-2 signaling is required for NK cells survival and cytokines production, a process requiring engagement of SHB [30], rising the hypothesis that interaction of KCTD9 with Cul3 and SHB may lead to degradation of SHB or SHB partners for turnover between NK cells activation and apoptosis.The exact mechanism underlying KCTD9 for NK cells function still needs to be clarified.

## Conclusions

In conclusion, our study described hydrodynamic delivery of plasmids expression shRNA against mKCTD9 attenuated liver damage and promoted mice survival of MHV-3 induced FHF. Such effect may be executed by efficient inhibition of mKCTD9 expression in NK cells and thus impaired NK cells activation which otherwise exacerbated liver damage. This research provides insight for novel strategy of target for therapeutic treatment viral fulminant hepatic failure.

## Additional files

Additional file 1: **Figure S1.** Survival of mice injected with 20 PFU and 100 PFU of MHV-3. Mice infected with 20 PFU of MHV-3 survived longer than those infected with 100 PFU of MHV-3. (TIF 9196 kb)

## Abbreviations

FHF: Fulminant hepatic failure
HBV-ACLF: HBV induced acute on chronic liver failure
KCTD9: Potassium channel tetramerisation domain containing 9
MHV-3: Mouse hepatitis virus strain 3
mKCTD9: Chronic hepatitis B
mKCTD9: Mouse KCTD9
PBMC: Peripheral blood mononuclear cell
shRNA: Short hairpin RNA
