# Supplementary figures and images for: Interference with KCTD9 inhibits NK cell activation and ameliorates fulminant liver failure in mice
Source: BMC Immunol. 2018 Jun 25;19:20. doi: 10.1186/s12865-018-0256-x (PMC6019787; doi:10.1186/s12865-018-0256-x)

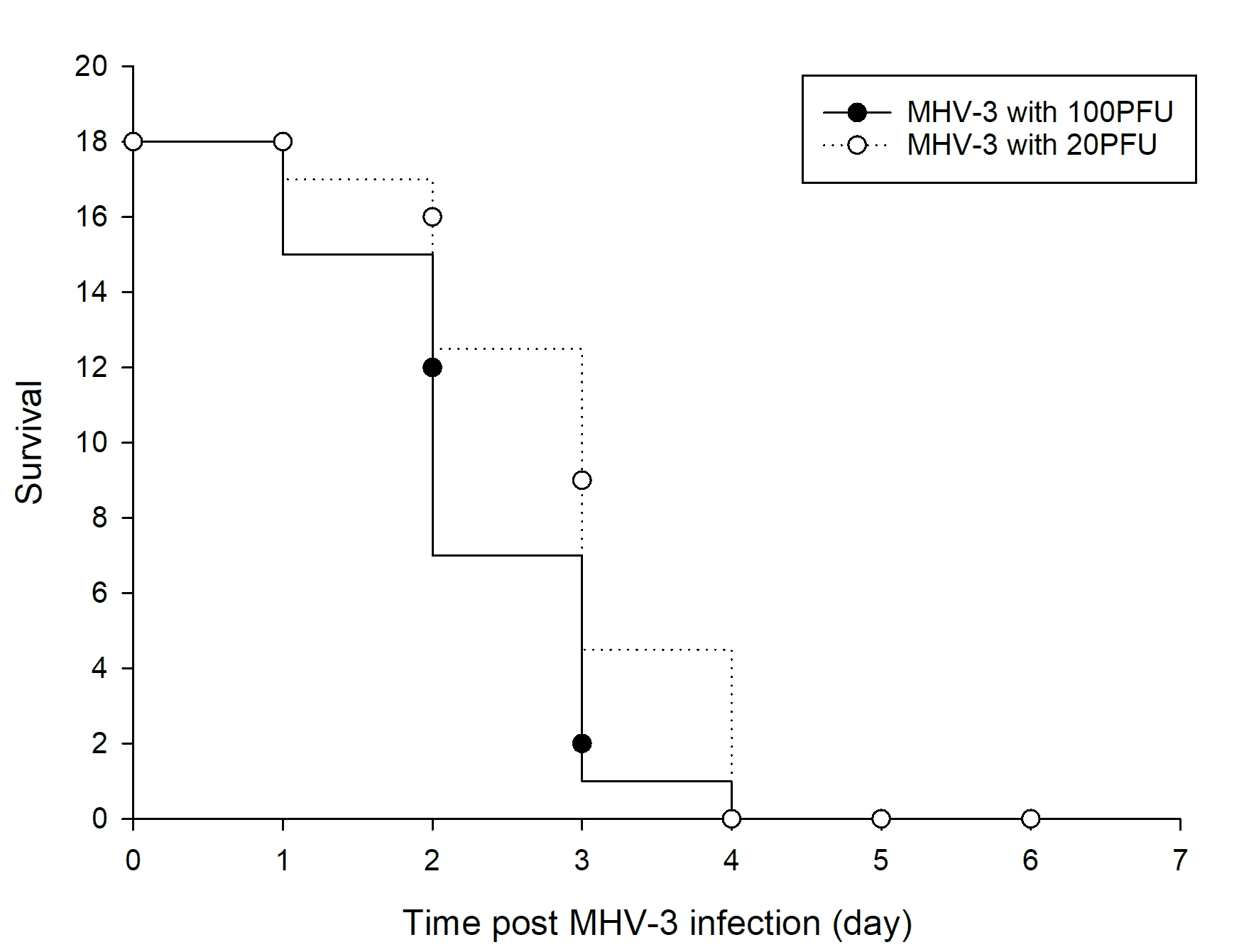

Supplement: Supplementary file 1 — Figure S1. Survival of mice injected with 20 PFU and 100 PFU of MHV-3. Mice infected with 20 PFU of MHV-3 survived longer than those infected with 100 PFU of MHV-3. (TIF 9196 kb) [file 12865_2018_256_MOESM1_ESM.tif]
